# The effect of leverage manipulation on real estate firms’ financial risk: Based on the interest conflicts perspective

**DOI:** 10.1371/journal.pone.0330709

**Published:** 2025-09-03

**Authors:** Liwen Huang, Liangxing Yu, Wei Huang

**Affiliations:** School of Business, Jishou University, Jishou, China; University of Almeria: Universidad de Almeria, SPAIN

## Abstract

From the perspective of interest conflicts, this study investigates the relationship between corporate leverage manipulation and financial risk using a sample of A-share listed real estate firms in China from 2009 to 2023. Employing a two-way fixed effects model, the main findings are as follows: (1) Leverage manipulation significantly increases the level of financial risk among real estate firms; (2) Mechanism analysis reveals a collusion effect between controlling shareholders and management, as well as between external auditors and management, both of which significantly amplify the impact of leverage manipulation on financial risk. These findings support the collusion effect hypothesis and reject the monitoring effect hypothesis; (3) Heterogeneity tests show that the impact of leverage manipulation on financial risk is more pronounced in non-state-owned enterprises, in firms dominated by transactional institutional investors, and in regions with lower reliance on land finance. This study uncovers the intrinsic link between leverage manipulation and financial risk in the real estate sector and provides important policy implications for regulators aiming to improve and standardize financial risk management in the industry.

## 1 Introduction

The real estate sector is a capital-intensive industry, requiring substantial funding to sustain operations across various phases, including development, construction, and other related activities. Given the relatively narrow financing channels in this sector, coupled with prolonged project cycles and slow capital turnover, it is inherently challenging for real estate enterprises to reduce leverage ratios through debt reduction, asset expansion, or a combination of both [1]. This structural vulnerability has led to significantly higher debt levels and increased risk exposure in the real estate sector compared to other industries.

**Fig 1:**
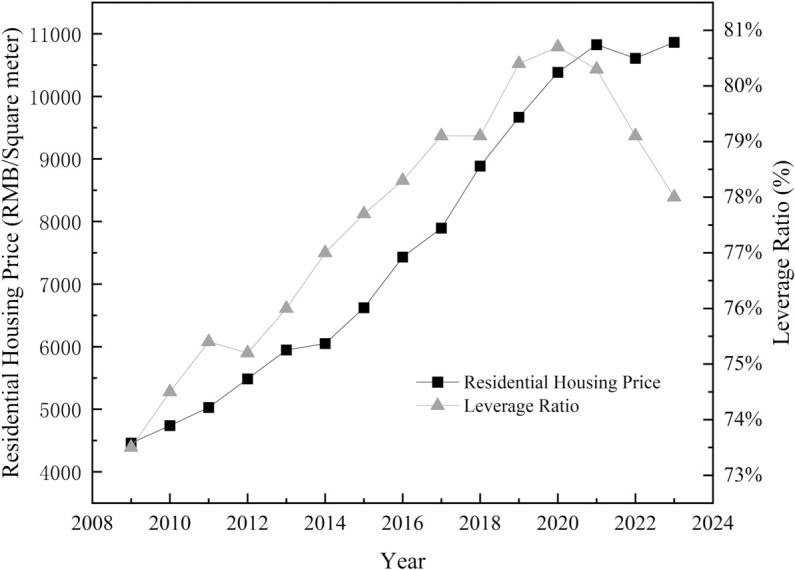
Average housing prices and leverage ratio in China’s real estate sector. Trends from 2009 to 2023 show a consistent rise in average housing prices (left Y-axis, RMB per square meter) and more volatile leverage ratios (right Y-axis, %). This divergence may indicate attempts by real estate firms to mask financial risk through leverage manipulation.

Following the 2008 global financial crisis, the Chinese government implemented a 4 trillion yuan stimulus package to stabilize economic growth. A portion of this package was allocated to real estate investments by enterprises through bank credit channels. In June 2015, the State Council introduced monetized compensation policies for urban redevelopment projects, which altered the monetary transmission mechanisms by channeling compensation funds directly into the property market.Although these policies were well-intentioned, they indirectly drove up housing prices and contributed to the continued rise in the leverage ratio of the real estate sector (as shown in Fig 1). Elevated leverage has been identified as a fundamental contributor to macroeconomic and financial system fragility [2]. The rapid accumulation of corporate debt, stemming from interconnected microeconomic actors, poses systemic threats to economic stability.

In response to these issues, the Central Economic Work Conference launched the “supply-side structural reforms” in December 2015, targeting overcapacity, inventory, leverage, costs, and weak growth areas, and this initiative marked the beginning of mandatory deleveraging in the real estate sector. By 2020, the “Three Red Lines” policy was introduced to tighten debt controls further, imposing quantitative thresholds on developers’ liability-to-asset ratios, liability-to-equity ratios, and cash-to-short-term-debt ratios to mitigate systemic risks. Confronted with constrained financing channels and regulatory pressures, some developers resorted to manipulation tactics to artificially suppress reported leverage ratios.

While such manipulation may temporarily improve balance sheet optics and facilitate access to short-term financing [3], it fails to address the underlying debt burdens. Instead, it masks financial vulnerabilities that jeopardize macroeconomic stability. Consequently, decoding the risk propagation mechanisms inherent in real estate leverage manipulation carries significant implications for safeguarding socioeconomic stability.

Based on the aforementioned research background, this study selects A-share listed real estate companies in Shanghai and Shenzhen stock exchanges from 2009 to 2023 as research samples, with data sourced from the CSMAR database. We measure leverage manipulation through three approaches: the expected XLT-LEVM method (*LEVM*), the expanded XLT-LEVM direct method (*ExpLEVM*), and the expanded XLT-LEVM indirect method (*ExpLEVMI*). The empirical results demonstrate that leverage manipulation in real estate enterprises significantly exacerbates financial risks, where a one-unit increase in *LEVM* results in a 0.204 increase in firm financial risk. These findings remain robust after addressing concerns about endogeneity and conducting robustness checks. In the mechanism analysis, we find that the management of real estate firms is embedded in a complex web of interest transfers with key stakeholders. Specifically, there is evidence of a collusion effect between controlling shareholders and management, as well as between external auditors and management, which significantly amplifies the impact of leverage manipulation on financial risk. Heterogeneity analyses indicate that non-state-owned enterprises and firms in regions with low fiscal land dependence display more substantial leverage manipulation incentives and consequent financial risks due to tighter financing constraints. Moreover, compared to firms dominated by stable institutional investors, the adverse effect of leverage manipulation on financial risk is more pronounced in companies with transaction-oriented institutional investors.

Compared with previous studies, this paper offers the following potential marginal contributions:

First, it investigates the impact of corporate leverage manipulation on financial risk within the real estate sector, a key sub-industry. This not only enriches the existing literature on the consequences of leverage manipulation but also expands the research scope on the underlying causes of financial risk in real estate firms.

Second, this study examines the mechanism through which stakeholders’ conflicts of interest influence the relationship between leverage manipulation and financial risk. By focusing on the internal role conflicts of stakeholders within real estate firms, this paper contributes to a better understanding of how such conflicts may affect governance effectiveness and risk outcomes.

Third, the study carries substantial practical implications. Specifically, in the context of a declining real estate market in China, exploring leverage manipulation behavior in real estate firms can provide early warning signals and risk prevention references for industry peers. It also offers valuable insights for policymakers seeking to build more effective regulatory frameworks and risk prevention mechanisms. Furthermore, the study enhances transparency of information for the public. It helps investors and consumers better identify risks in the real estate sector, thereby contributing to the healthy development of capital markets.

The remainder of this paper is organized as follows: Sect 2 reviews relevant literature on leverage manipulation and financial risk; Sect 3 develops the theoretical framework and proposes research hypotheses; Sect 4 details the research design, including data sources, variable definitions, and model specifications; Sect 5 presents baseline regression results and robustness tests; Sect 6 conducts mechanism and heterogeneity analyses; Sect 7 concludes with research findings and limitations.

## 2 Literature review

### 2.1 Research on financial risk

Existing research has formed a multi-layered understanding of the formation mechanism of corporate financial risks, which is mainly reflected in the following two aspects: At the macro level, studies emphasize the impact of economic policy uncertainty on operational strategies [4,5]. Some scholars have also investigated the effects of extreme weather on corporate financial conditions [6,7]. At the micro level, scholars have focused on the influences of factors such as corporate governance, board characteristics, CEO characteristics, and corporate liquidity on corporate financial risks [8–13]. Notably, excessive leverage ratios are widely recognized as a critical catalyst for financial distress [14,15]. Although the trade-off theory posits that firms can mitigate financial risks by optimizing capital structures[16], empirical evidence reveals pervasive intentional underreporting of book leverage. For instance, Kraft(2015) [17] demonstrated how U.S. firms exploit operating leases to conceal liabilities in credit rating processes. Similarly, Mills and Newberry (2005), Callahan et al. (2012), and Yue and Wu (2009) [18–20] identified structural financing techniques, such as off-balance-sheet hybrid debt instruments and convertible bonds, that are used to inflate equity artificially. Christensen and Nikolaev (2013) [21] further revealed that the fair value revaluation of non-financial assets helps firms avoid debt covenant violations by lowering reported leverage ratios, thereby enhancing loan accessibility.

### 2.2 Research on leverage manipulation

Leverage manipulation refers to the practice whereby corporate managers or controlling shareholders artificially modify on-balance-sheet leverage ratios through unconventional means—such as off-balance-sheet liabilities non-share-real-debts—in order to navigate financing constraints or regulatory pressure. This behavior exploits the flexibility of accounting standards and regulatory loopholes, without substantively reducing actual debt risk.

Braithwaite (1985) [22] defines such behavior, in which individuals with high social status and professional or managerial roles exploit their positions for short-term private gains, as white-collar crime. For instance, managers may engage in rent-seeking activities to conduct earnings management or use tools such as stock price manipulation and insider trading to obtain short-term private benefits [23–25]. Leverage manipulation is considered one form of white-collar crime.

As for the motivation behind managerial leverage manipulation, prior studies suggest that external institutional pressures often drive such behavior. When firms face financing crowding-out effects caused by local government debt expansion [26], cash flow pressure from environmental tax reform [27], or rising labor costs due to increases in minimum wage standards [28], their financing, regulatory, and labor costs tend to rise across the board. In such contexts, management is more inclined to use leverage manipulation as a means of risk shifting.

Regarding the methods of leverage manipulation, Huang(2005) [29] finds that some firms understate asset depreciation (or amortization) and impairment provisions in order to reduce their reported debt-to-asset ratios. Daley and Vigeland (1983) [30], Wang et al. (2011) [31] identified that inflated R&D capitalization rates serve as a common tactic to deflate reported debt levels. Additionally, firms manipulate non-recurring gains or losses to obscure proper leverage [32,33]. Notably, firm-specific characteristics correlate with manipulation tactics. Cotter and Zimmer (1995) [34] observed that firms with declining operating cash flows revalue assets to boost collateral values, which signals borrowing capacity. Mills and Newberry (2005) [18] found that firms with lower credit ratings or higher leverage tend to utilize off-balance-sheet financing and hybrid debt instruments. Furthermore, highly regulated or financially constrained firms often engage in asset securitization and fair value engineering to mask excessive leverage [35,36]. In response to these practices, Xu et al. (2020) [37] categorize corporate tactics that are used to obscure actual leverage levels and formally define them as leverage manipulation disproportionately.

Internally, optimizing corporate governance structures, such as embedding Party organization oversight [38], shareholder governance via mixed-ownership reforms [39,40], and digital transformation enhancing transparency [41,42], is reshaping financial decision-making logics. Externally, audit supervision’s signaling role [1], institutional investor activism [43,44], and media-driven reputational constraints [45] collectively form a multi-dimensional risk mitigation framework.

Research on the economic consequences remains underdeveloped relative to studies on causality and governance. However, existing work confirms that leverage manipulation distorts resource allocation efficiency [46], elevates default probabilities [47], and amplifies macroeconomic volatility through risk contagion [48].

Despite extensive research on the financial risk determinants of firms, the consequences of leverage manipulation remain underexplored in the real estate sector, a systemically critical industry with complex supply chains and diverse stakeholders. Leverage manipulation distorts accounting transparency by decoupling reported and actual leverage ratios, thus elevating information asymmetry risks. Stakeholders who confront such opacity often make irrational decisions, which destabilize capital markets and societal welfare. Clarifying how real estate leverage manipulation propagates financial risks is, therefore, imperative for regulatory design and economic stability.

## 3 Theoretical analysis and research hypothesis

### 3.1 Leverage manipulation and financial risk in real estate firms

Leverage manipulation by real estate firms is highly likely to exacerbate their financial risk. According to transaction cost theory, firms incur certain costs in market transactions, including information search costs and contracting costs. To reduce these transaction costs—particularly financing and compliance costs—firms tend to obscure the clarity of information disclosure and avoid transparency [49,50], thereby reducing external stakeholders’ ability to accurately assess their actual leverage levels. This information asymmetry leads to market failure, making it difficult for external investors and creditors to properly evaluate the firm’s debt-servicing capacity and actual financial condition [51]. As a result, firms receive financing at an underestimated cost of risk, allowing them to not only lower financing expenses but also bypass policy and regulatory constraints, thereby reinforcing leverage manipulation. However, once external economic conditions shift, these firms face a high risk of cash flow disruption and may fall into severe financial distress.

Cohen and Felson (1979) [52] argue that three conditions are necessary for criminal behavior to occur: a motivated offender, a suitable target, and the absence of effective guardianship. Within the Chinese context, leverage manipulation by real estate firms—a form of white-collar crime—is a widespread phenomenon. First, managerial motivation is primarily driven by the allure of short-term economic gains. To maintain liquidity and business continuity, executives often resort to increasingly aggressive leverage manipulation strategies to enhance short-term financial performance. This enables them to secure rapid access to capital, support ongoing expansion, and maximize personal benefits such as executive compensation and equity incentives [53].

Second, a suitable target. Since 2009, the Chinese central government has introduced a series of credit and financing regulations that have significantly raised compliance costs and financing difficulties for real estate firms. In response, many firms conceal part of their debt through off-balance-sheet financing and other means to avoid detection by regulators and creditors. Due to the inherently complex financial structures of real estate firms—often involving project-based financing, perpetual bonds, minority interests, and other hybrid instruments—their financial reports provide considerable room for manipulation, making them ideal targets for leverage manipulation by corporate insiders.

A typical example is Evergrande Group. To cope with a tightening financing environment while pursuing large-scale development projects and continuing its business expansion, Evergrande issued a significant volume of perpetual bonds between 2013 and 2016. These were recorded under owner’s equity, effectively lowering the company’s reported debt-to-asset ratio. This “non-share-real-debts” arrangement enabled the firm to evade regulatory scrutiny, making it a textbook case of how real estate firms serve a “suitable targets” for such financial manipulation.

Lastly, weak regulatory oversight stems from China’s unique policy environment. As a pillar industry, real estate is closely tied to local economic growth and the performance evaluations of local governments [54]. For a long time, local government revenue has heavily depended on land sales and real estate development [55]. The promotion system for local officials essentially drives this. Given that officials typically serve short terms (usually 3–5 years), prioritizing short-term economic performance by favoring real estate development becomes a rational choice [56]. Even when local governments are aware of excessive leverage and financial risks in real estate firms, they often look the other way for the sake of maintaining favorable political performance metrics. This lax regulatory environment provides real estate firms with the "room to maneuver" in manipulating leverage. It allows them to expand their debt levels with minimal market scrutiny continuously.

This combination of information asymmetry and regulatory leniency not only increases corporate financial risk but also contributes to systemic failures in regulation and the market. As leverage risks accumulate over time, they may eventually trigger widespread instability within the real estate sector. Based on the above reasoning, this study proposes the following hypothesis:

**H1.** The degree of leverage manipulation in real estate firms is positively associated with their financial risk exposure.

### 3.2 Mechanism analysis

The preceding analysis demonstrates that leverage manipulation exacerbates corporate financial risks. To clarify the underlying logical relationships, the following mechanism analysis is conducted from the two perspectives of “monitoring effects” and “collusion effects.”

#### 3.2.1 Leverage manipulation, major shareholders, and financial risk.

Leverage manipulation inherently stems from compounded agency problems and information asymmetry. Based on Jensen and Meckling’s Principal-Agent Theory [57], the separation of ownership and control generates dual agency dilemmas: managerial opportunism at the expense of shareholder value, and controlling shareholders expropriating minority interests.

As monitors, large shareholders can constrain managerial opportunism through “voice” and “exit” mechanisms. The voice mechanism involves vetoing aggressive off-balance-sheet financing proposals via board representation or demanding third-party audits of hidden liabilities [58]. The exit mechanism signals risk through share divestments, pressuring management to recalibrate financial strategies [59]. However, monitoring efficacy hinges on Major shareholders’ governance incentives and informational capacity. In real estate firms with dispersed ownership, major shareholders often lack the motivation to bear high monitoring costs, resulting in free-rider problems [60], which incentivizes managerial leverage manipulation for self-interest.

When major shareholders themselves benefit from leverage manipulation, monitoring incentives yield collusive motives. China’s real estate sector, characterized by hyper-concentrated ownership, fosters such role distortions. Major shareholders typically pursue dual objectives: shared benefits from firm value appreciation and private benefits extracted via control rights. To maximize private gains, major shareholders may assist management in evading regulatory scrutiny through complex ownership chains, while management manipulates leverage to sustain stock price stability via “low-leverage illusions.” This collusive equilibrium relies on mutual information monopolies: major shareholders suppress critical disclosures through board dominance, while management exploits accounting flexibility to adjust discretionary accruals [61], creating bilateral informational barriers.

Theoretical remedies, such as equity incentives and performance-linked contracts, designed to align interests, become distorted under concentrated ownership. In Chinese real estate firms [62], their effectiveness depends on power dynamics between major shareholders and management. When collusion prevails, compensation contracts morph into covert collusive pacts: managers facilitate off-balance-sheet financing in exchange for accelerated option vesting while major shareholders engage in earnings management to meet vesting thresholds [63,64]. This incentive distortion reflects the interplay of agency theory and information asymmetry, where major shareholders’ informational advantages lubricate collusion rather than enabling oversight, and managers’ private information becomes a bargaining chip [65–67]. Based on this analysis, we propose competing hypotheses:

**H2a.** Major shareholders in real estate firms exert monitoring effects, significantly mitigating the positive impact of leverage manipulation on financial risk.

**H2b.** Collusion between major shareholders and management amplifies the positive association between leverage manipulation and financial risk.

#### 3.2.2 Leverage manipulation, external auditing, and financial risk.

The covert nature of leverage manipulation acts as a catalyst for the intertemporal accumulation of financial risks, whereas external auditing mitigates such risks by resolving information asymmetry. Drawing on Akerlof’s (1978) [51] “market for lemons” framework, information-disadvantaged parties (e.g., investors, creditors) systematically underestimate market quality due to their inability to discern firms’ accurate risk profiles, triggering adverse selection and moral hazard. External auditing addresses this dilemma through dual mechanisms.

First, the information verification mechanism enables industry-specialized auditors to precisely identify leverage manipulation tactics—including off-balance-sheet liabilities and accounting mismatches in perpetual debt instruments—by leveraging their expertise in real estate project cycles and financing structures [68]. This professional capability essentially reprocesses private corporate information into auditable signals, constraining managerial opportunism rooted in informational monopolies. Second, the reputational disciplining mechanism compels auditors to demand incremental disclosures on material financing activities to protect their reputational capital [69]. Such disclosures operate as proactive signals [70], correcting information asymmetry by revealing risk-pricing cues to investors and regulators. When audit opinions serve as credibility benchmarks for capital market access, real estate firms face heightened incentives to curb the intensity of leverage manipulation to preserve refinancing capacity, ultimately reducing corporate risk exposure.

An alternative perspective posits that auditor independence faces endogenous challenges stemming from the principal-agent dynamics inherent in client-auditor contractual relationships. First, reputation mechanisms, which are the core disciplinary force in auditing, require mature institutional ecosystems to function effectively. In China’s underdeveloped reputation-pricing market, economic returns from audit services frequently override ethical constraints, incentivizing accommodative strategies to retain clients and eroding gatekeeping efficacy. A more profound structural paradox exists: Auditors are legally mandated to serve as public-interest watchdogs, but are economically dependent on their audit clients. The “athletes-hiring-referees” dynamic systematically misaligns incentives, prioritizing client satisfaction over fiduciary duties [71].

Furthermore, the cumulative risk effects of leverage manipulation exacerbate these tensions. Unlike single-period earnings manipulation, leverage manipulation compounds risks by creating layered off-balance-sheet liabilities and pseudo-equity instruments. Real estate firms exemplify this pathology: to sustain capital market confidence amid high leverage, developers forge implicit collusion contracts with auditors—tacitly permitting complex financial engineering to circumvent the CSRC’s Disclosure Rule No. 14 on financing restrictions, while reciprocating with abnormal fees [72]. Although fully capable of detecting manipulation techniques, auditors in distorted relationships repurpose their expertise into “technical compliance” enablers. This duality motivates competing hypotheses:

**H3a.** External auditors’ monitoring function significantly mitigates the positive association between leverage manipulation and financial risk in real estate firms.

**H3b.** Collusion between external auditors and real estate firms amplifies the positive association between leverage manipulation and financial risk.

## 4 Research design

### 4.1 Sample selection

This study focuses on Chinese A-share listed real estate companies from 2009 to 2023. The selection of this timeframe is grounded in the significant impact of the 2008 global financial crisis on China’s economy. In response to the crisis, the Chinese government introduced a series of large-scale economic stimulus measures. These interventions had a profound influence on firms’ leverage levels and financing behaviors. As a result, 2009 marked a critical turning point in the recovery of real estate firms, providing a meaningful context for studying the emergence and intensification of high-leverage and leverage manipulation practices. During this period, the industry experienced a wave of recovery and expansion, making it a particularly relevant window for research on leverage manipulation.

To ensure data quality, this study excludes firms designated as ST and *ST to minimize potential bias from companies with extreme financial distress. Continuous variables are winsorized at the 1% level to reduce the influence of outliers, and observations with substantial missing values are excluded. Interpolation methods are applied to fill in gaps for core variables, helping to maintain both the representativeness and completeness of the dataset. The final sample consists of 1,316 firm-year observations. Firm-level financial data are sourced from the CSMAR database, while macroeconomic data are obtained from the EPS database and the National Bureau of Statistics of China.

### 4.2 Variable definitions

#### 4.2.1 Explained variables.

Drawing on Ohlson’s (1980) [73] research, this study uses the O-Score model to calculate the O-index as an indicator of corporate financial risk levels (*Risk*). A higher O-value signifies a greater probability of corporate bankruptcy. The specific calculation formula is as follows:

$${Risk}_{i,t}=\;-1.32-0.407\cdot {SIZE}_{i,t}+6.03\cdot {TLTA}_{i,t}-1.43\cdot {WCTA}_{i,t}\;+0.0757\cdot {CLCA}_{i,t}-2.37\cdot {NITA}_{i,t}-1.83\cdot {FUTL}_{i,t}\;+0.285\cdot {INTWO}_{i,t}-1.72\cdot {OENEG}_{i,t}+0.521\cdot {CHIN}_{i,t}$$ (1)

Here, *SIZE* is computed as the natural logarithm of total assets adjusted by price indices. *TLTA* is the ratio of total liabilities to total assets, measuring overall financial leverage. *WCTA*, the ratio of working capital to total assets, reflects short-term liquidity. *CLCA*, the ratio of current liabilities to current assets, assesses immediate solvency. *NITA*, calculated as net income divided by total assets, evaluates profitability. *FUTL*, the ratio of operating cash flow to total liabilities, gauges cash flow adequacy. *INTWO* is a dummy variable that equals 1 if a firm has reported negative net income for two consecutive years, indicating sustained financial distress. *OENEG* is a binary indicator set to 1 when a firm’s total liabilities exceed total assets, signaling insolvency risk; otherwise, it is 0. *CHIN* captures the change in net income from the previous year to the current year, normalized by the sum of their absolute values, reflecting profitability dynamics.

#### 4.2.2 Explanatory variables.

Adopting the XLT-LEVM method under the expected model framework [37], leverage manipulation intensity (*LEVM*) is quantified as:

$${LEVM}_{i,t}=({DEBTB\_TOTAL}_{i,t}+{DEBT\_OB}_{i,t}+{DEBT\_NSRD}_{i,t})/\;\;({ASSETB\_TOTAL}_{i,t}+{DEBT\_OB}_{i,t})-{LEVB}_{i,t}$$ (2)

Where *LEVM* measures the extent of corporate leverage manipulation based on the XLT-LEVM method (expected model framework). DEBTB_TOTAL represents a firm’s total on-balance-sheet liabilities, DEBT_OB captures off-balance-sheet liabilities, and DEBT_NSRD quantifies nominal equity instruments with actual debt obligations (non-share-real-debts). ASSETB_TOTAL is the total book assets, and *LEVB* is defined as the book leverage ratio. Higher *LEVM* values correspond to more severe leverage manipulation. The expected model method estimates the DEBT_OB and the DEBT_NSRD.

For robustness, leverage manipulation is re-estimated using two variants of the expanded XLT-LEVM method: *ExpLEVM* (direct method) and *ExpLEVMI* (indirect method).

$${ExpLEVM\_l}_{i,t}=\left({DEBTB\_TOTAL}_{i,t}+{DEBT\_OB}_{i,t}+{DEBT\_NSRD}_{i,t}\right)/\;({ASSETB\_TOTAL}_{i,t}+{DEBT\_OB}_{i,t}-{DM\_ASSET}_{i,t}\;\;-{RDM\_ASSET}_{i,t})-{LEVB}_{i,t}$$ (3)

$${ExpLEVMI\_l}_{i,t}=\left({DEBTB\_TOTAL}_{i,t}+{DEBT\_OB}_{i,t}+{DEBT\_NSRD}_{i,t}\right)/\;\left({ASSETB\_TOTAL}_{i,t}+{DEBT\_OB}_{i,t}-{DA}_{i,t}\right)-{LEVB}_{i,t}$$ (4)

*ExpLEVM* and *ExpLEVMI* measure leverage manipulation under the expanded XLT-LEVM framework, corresponding to the direct and indirect methods, respectively. In Eq (3), DM_ASSET represents the overstatement of total assets through inflated fixed asset depreciation, while RDM_ASSET captures asset overvaluation via the capitalization of research and development expenditures. In Eq (4), discretionary accruals(*DA*) are estimated as a proxy for earnings manipulation. Other variables match Eq (2).

#### 4.2.3 Control variables.

To account for confounding factors, we include ownership type (*Soe*), firm size (*Size*), revenue growth (*Growth*), inventory ratio (*Inv*), CEO duality (*Dual*), managerial ownership (*Msl*), independent director ratio (*Indep*), board size (*Board*), current ratio (*Liquid*), profitability (*Roa*), market valuation (*TobinsQ*), and loss status (*Loss*), consistent with prior studies. Definitions and operationalization details are summarized in Table 1.

**Table 1:** Variable definitions.

Variable type	Variable name	Symbol	Definition/Measurement
Dependent variable	Financial Risk	*Risk*	O-score index; the higher the value, the greater the likelihood of corporate bankruptcy.
Independent variable	Leverage Manipulation	*LEVM*	Estimated using the expected model approach (XLT - LEVM method).
Control variables	Firm Size	*Size*	Natural logarithm of total assets at year-end.
	Liquidity Ratio	*Liquid*	Current assets divided by current liabilities.
	Managerial Ownership	*Msl*	Shares held by executives and directors / total shares outstanding.
	Board Independence	*Indep*	Number of independent directors / total board size.
	Tobin’s Q	*TobinQ*	Market value / total assets.
	Profitability	*Roa*	Net income / total assets.
	Ownership Nature	*Soe*	1 if the firm is state-owned, 0 otherwise.
	Board Size	*Board*	Natural logarithm of the total number of board members.
	CEO Duality	*Dual*	1 if the CEO also serves as board chair, 0 otherwise.
	Growth	*Growth*	Growth rate of operating revenue.
	Inventory Ratio	*Inv*	Ending balance of inventories / total assets.
	Loss Indicator	*Loss*	1 if the firm reports a loss in the current year, 0 otherwise.

### 4.3 Model specification

To empirically validate Hypothesis H1, we estimate the following regression model to assess how leverage manipulation influences financial risk in real estate firms:

$${Risk}_{i,t}=\alpha_{0}+\alpha_{1}{LEVM}_{i,t}+\alpha_{2}{Controls}_{i,t}+\mu_{i,t}+\eta_{i,t}+\varepsilon_{i,t}$$ (5)

Here, *Risk* denotes the financial risk measure for firm i in year t; *LEVM* captures the intensity of leverage manipulation; and Controls represents a vector of firm-level covariates detailed in Table 1. The model incorporates firm-fixed effects (*μ*) to account for unobserved heterogeneity across firms and year-fixed effects (*η*) to control for temporal shocks. Standard errors are clustered at the firm level to address residual autocorrelation.

A statistically significant positive estimate of $\alpha_{1}$ would support Hypothesis H1, indicating that leverage manipulation amplifies firm financial risk exposure. This specification isolates the marginal effect of leverage manipulation while mitigating confounding through rigorous econometric controls.

## 5 Empirical analysis

### 5.1 Descriptive statistics

Table 2 presents descriptive statistics. The financial risk measure (*Risk*) for real estate firms ranges from -11.565 to 0, with a standard deviation of 1.555, indicating substantial heterogeneity in financial vulnerability across Chinese property developers. A subset of firms exhibits extreme risk exposures, highlighting sector-wide fragility. Leverage manipulation (*LEVM*) demonstrates pronounced dispersion, spanning from -18.521 to 30.562, which may reflect divergent strategic orientations that Firms pursuing aggressive expansion strategies likely drive the upper tail of manipulation intensity. Distributions of the remaining variables are reported in Table 2.

**Table 2:** Descriptive statistics.

Variable	Obs	Mean	Std. Dev.	Min	Max
*Risk*	1316	-7.414	1.555	-11.565	0.000
*LEVM*	1316	0.196	1.399	-18.521	30.562
*Size*	1316	23.475	1.422	20.674	27.511
*Liquid*	1316	1.942	0.901	0.407	6.131
*Msl*	1316	0.002	0.065	0.000	0.368
*Indep*	1316	37.918	5.714	30.000	57.14
*TobinQ*	1316	1.308	0.530	0.782	4.034
*Roa*	1316	0.025	0.034	-0.095	0.125
*Soe*	1316	0.604	0.489	0.000	1.000
*Board*	1316	2.134	0.184	1.609	2.565
*Dual*	1316	0.115	0.319	0.000	1.000
*Growth*	1316	2.814	6.529	-0.791	44.805
*Inv*	1316	0.504	0.225	0.000	0.862
*Loss*	1316	0.066	0.249	0.000	1.000

Diagnostic tests confirm model robustness. The Hausman test for Eq (5) yields a p-value of 0.000, statistically justifying the fixed-effects specification over random effects. Variance Inflation Factor (VIF) analysis reveals no severe multicollinearity concerns, with individual VIFs ranging from 1.06 to 1.59 and a mean VIF of 1.28, well below the conventional threshold of 5.0. These diagnostics validate the empirical design’s capacity to isolate the causal relationship between leverage manipulation and financial risk.

### 5.2 Baseline regression

Table 3 presents the benchmark regression results. Columns (1) and (2) do not control for fixed effects. Specifically, Column (1) includes only the dependent variable, where the coefficient of leverage manipulation (*LEVM*) is significantly positive at the 5% level of significance. Column (2) adds control variables, and the regression coefficient of leverage manipulation (*LEVM*) becomes significantly positive at the 1% level.

**Table 3:** Baseline regression.

Variables	(1)	(2)	(3)	(4)
	*Risk*	*Risk*	*Risk*	*Risk*
*LEVM*	0.169^**^	0.241^***^	0.172^**^	0.204^***^
	(2.499)	(3.403)	(2.275)	(2.942)
*Size*		-0.223^***^		-0.474^***^
		(-4.206)		(-3.403)
*Liquid*		-0.609^***^		-0.420^***^
		(-7.569)		(-4.908)
*Msl*		4.363^**^		7.218^***^
		(2.010)		(2.996)
*Indep*		-0.021^**^		0.009
		(-2.040)		(0.690)
*TobinQ*		-0.051		-0.368^*^
		(-0.400)		(-2.342)
*Roa*		-19.880^***^		-23.225^***^
		(-10.469)		(-13.045)
*Soe*		0.190		-0.365^*^
		(1.395)		(-2.311)
*Board*		-0.297		-0.349
		(-0.685)		(-0.723)
*Dual*		-0.080		0.006
		(-0.398)		(0.058)
*Growth*		0.001		-0.000
		(0.303)		(-0.019)
*Inv*		0.979^***^		0.864^***^
		(2.850)		(2.660)
*Loss*		-0.729^***^		-0.589^***^
		(-5.318)		(-4.976)
*Constant*	-7.447^***^	0.324	-7.448^***^	5.630
	(-77.705)	(0.198)	(-501.501)	(1.630)
Year FE	No	No	Yes	Yes
Firm FE	No	No	Yes	Yes
N	1316	1316	1316	1316
Adj. R^2^	0.022	0.346	0.333	0.544

*Notes:*  , ^**^, ^***^ indicate significance at the 10%, 5%, and 1% levels, respectively. t-values in parentheses are clustered at the firm level.

Columns (3) and (4) further control for time and firm fixed effects. Column (3) shows the regression results without control variables, where the *LEVM* coefficient is significantly positive at the 5% level. After adding control variables in Column (4), the *LEVM* coefficient is 0.204 and significantly positive at the 1% level, with a slight increase compared to the coefficient without control variables. The results consistently affirm that leverage manipulation exacerbates financial risk in real estate firms, thereby providing empirical support for Hypothesis H1.

### 5.3 Endogeneity test

#### 5.3.1 Instrumental variable method.

To address potential endogeneity concerns such as reverse causality and omitted variables, this study employs a two-stage least squares (2SLS) approach with instrumental variables (IVs). Drawing on Greif and Tabellini (2017) and Pan et al. (2019) [74,75], clan culture is selected as a primary IV. Specifically, clan culture intensity is measured as the natural logarithm of the number of genealogical records (from the Qing Dynasty to post-1990) per capita in a given region.

From a correlation perspective, clan culture, a key informal institution, can alleviate corporate financing constraints, thus reducing leverage manipulation. The trust within clan networks, built on kinship and geography, enables “relationship-based financing,” helping firms access informal financial resources like private loans and rotating savings [76]. Also, the moral norms of clan culture, such as collective honor and generational responsibility [77], can restrain managerial self-interest and motivate firms to opt for transparent financing over risky leverage manipulation [78]. From an exogeneity perspective, the spatial distribution of clan culture is primarily influenced by pre-modern factors, such as historical migration routes and geographical isolation [79]. The clan intensity measured by genealogy compilation during the Ming and Qing Dynasties has historical lags, predating contemporary corporate financial decision-making. Therefore, it is not directly linked to current firm financial risks. Notably, since clan culture is a cross-sectional variable incompatible with panel data structures, the instrument is constructed as the interaction between clan culture intensity and time dummies (*Clan*). For robustness, a secondary instrument (LEVM_cubed) is derived from the cubic term of leverage manipulation deviations from their mean, following Huang et al. (2018) [80].

As shown in column (1) of Table 4, the first-stage regression confirms the instruments’ relevance: both *Clan* and LEVM_cubed exhibit statistically significant coefficients, with an F-statistic of 26.95 far exceeding the Stock-Yogo critical value of 19.93 for a 10% maximal bias. Additionally, the Hansen J-test (p = 0.320) validates instrument exogeneity. Column (2) presents second-stage results, where leverage manipulation (*LEVM*) remains significantly positive at the 1% level, indicating that its risk-amplifying effect persists after addressing endogeneity, thereby reinforcing the robustness of the baseline findings.

**Table 4:** Endogeneity test I.

Variables	Instrumental Variable Testing		Heckman Two-Stage	
	(1) First stage	(2) Second stage	(3) First stage	(4) Second stage
	*LEVM*	*Risk*	LEVM_Dum	*Risk*
*LEVM*		0.278^***^		0.299^***^
		(-4.86)		(-6.465)
*Clan*	-0.000^*^		-0.000^**^	
	(-1.685)		(-1.972)	
LEVM_cubed	0.001^***^			
	(-7.206)			
*IMR*				-0.374
				(-0.567)
*Size*	0.004	-0.471^***^	0.084	-0.419^**^
	(0.055)	(-3.314)	(0.647)	(-2.444)
*Liquid*	0.031	-0.434^***^	0.019	-0.427^***^
	(0.608)	(-4.950)	(0.263)	(-4.648)
*Msl*	2.316	6.721^***^	3.295^*^	5.322^**^
	(1.186)	(2.633)	(1.716)	(2.063)
*Indep*	0.001	0.009	0.040^**^	0.011
	(0.14)	(0.669)	(2.561)	(0.674)
*TobinQ*	-0.050	-0.354^**^	0.101	-0.253
	(-0.363)	(-2.180)	(0.614)	(-1.480)
*Roa*	2.270^**^	-23.538^***^	1.275	-23.647^***^
	(2.010)	(-13.219)	(0.636)	(-16.041)
*Soe*	-0.057	-0.352^**^	0.123	-0.319
	(-0.331)	(-2.178)	(0.470)	(-1.544)
*Board*	0.079	-0.353	0.506	0.232
	(0.278)	(-0.735)	(0.745)	(0.409)
*Dual*	0.033	-0.002	0.207	-0.020
	(0.357)	(-0.017)	(1.220)	(-0.154)
*Growth*	-0.007^**^	0.000	-0.008	0.001
	(-2.188)	(0.075)	(-0.882)	(0.126)
*Inv*	0.111	0.859^**^	-0.293	1.001^**^
	(0.414)	(2.577)	(-0.694)	(2.580)
*Loss*	0.108	-0.604^***^	0.509^**^	-0.653^***^
	(0.940)	(-5.118)	(2.261)	(-3.021)
*Constant*	0.280	-	-3.646	2.920
	(0.135)	-	(-0.890)	(0.763)
Year FE	Yes	Yes	Yes	Yes
Firm FE	Yes	Yes	Yes	Yes
Observations	1301	1301	1189	915
Adj. R^2^	0.615	0.325	-	0.627

#### 5.3.2 Heckman two-stage model.

To address potential selection bias, this study employs the Heckman two-stage correction method. In the first-stage Probit model, we construct a binary variable (LEVM_Dum) based on Xu and Song’s approach [2], which takes a value of 1 if the firm’s leverage manipulation measure (*LEVM*) is strictly positive, indicating the presence of upward leverage adjustment; otherwise, it takes a value of 0. The instrumental variable (*Clan*) is utilized to estimate the Inverse Mills Ratio (*IMR*), which is then included in the second-stage regression to mitigate sample selection bias.

Columns (3) and (4) of Table 4 presents Heckman regression results. Column (3) shows a significantly negative coefficient for the *IV* at the 5% level, confirming that clan culture has a material influence on leverage manipulation decisions. The statistically insignificant *IMR* in the second stage indicates negligible sample self-selection bias. Crucially, the coefficient for *LEVM* remains significantly positive at the 1% level, robustly affirming our core findings against concerns about selection bias.

#### 5.3.3 Propensity score matching.

Although Eq (5) incorporates extensive control variables, residual sample selection bias may persist due to unobserved heterogeneity. For instance, firms engaging in leverage manipulation may exhibit distinct strategic preferences, such as aggressive expansion tendencies or managerial risk appetite, that independently influence financial risk, confounding the causal interpretation of leverage manipulation effects. To address this, we implement Propensity Score Matching (PSM) to isolate the treatment effect of leverage manipulation.

**Fig 2:**
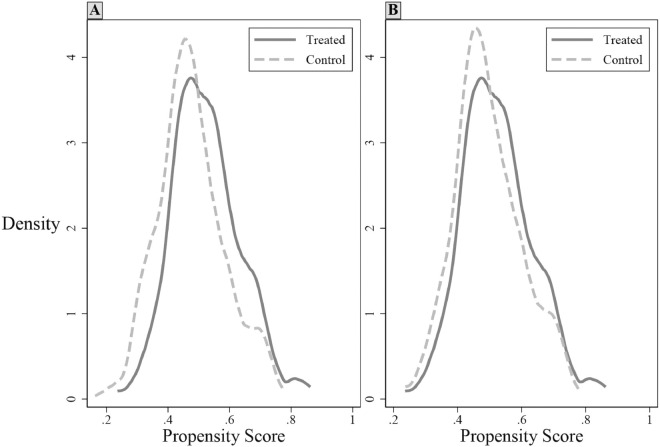
Kernel density distribution plot. Panel A shows pre-matching distributions with covariate imbalance. Panel B shows post-matching distributions with overlapping curves, confirming covariate balance and common support after propensity score matching.

The sample is partitioned into treatment and control groups based on the median leverage manipulation (*LEVM*), with firms above the median assigned to the treatment group (Treated) and those below the median to the control group (Control). Using all control variables from Eq (5) as covariates, we perform nearest-neighbor matching with replacement (1:2 ratio) and a caliper of 0.01. Post-matching diagnostics in Table 5 confirm covariate balance, with standardized biases below 10% and no significant intergroup differences, satisfying the balancing property. Fig 2 demonstrates enhanced overlap in kernel density distributions between groups, fulfilling the common support assumption. Re-estimating the model on matched samples, Table 6 column (1) demonstrates that the coefficient on treated firms’ leverage manipulation remains significantly positive, affirming the robustness of our core findings.

**Table 5:** Balance test.

Variables	Unmatched	Mean		%bias	%reduct |*bias*|	T-test	
	Matched	Treated	Control			t-value	𝐩>|𝐭|
*Size*	U	23.530	23.420	7.3	65.50	1.330	0.183
	M	23.570	23.610	–2.5		–0.440	0.658
*Liquid*	U	1.893	1.992	–11.0	88.50	–2.000	0.045
	M	1.883	1.871	1.3		0.230	0.817
*Msl*	U	0.026	0.014	18.2	85.30	3.310	0.001
	M	0.020	0.022	–2.7		–0.470	0.636
*Indep*	U	38.140	37.700	7.8	21.30	1.420	0.157
	M	38.160	37.810	6.1		1.080	0.278
*TobinQ*	U	1.307	1.309	–0.4	–917.30	–0.070	0.948
	M	1.293	1.312	–3.7		–0.650	0.518
*Roa*	U	0.029	0.022	20.9	69.70	3.780	0.000
	M	0.028	0.025	6.3		1.140	0.256
*Soe*	U	0.614	0.594	4.0	–2.00	0.730	0.464
	M	0.626	0.606	4.1		0.740	0.457
*Board*	U	2.142	2.125	9.2	86.50	1.670	0.096
	M	2.143	2.145	–1.2		–0.230	0.820
*Dual*	U	0.122	0.108	4.3	94.30	0.780	0.437
	M	0.119	0.120	–0.2		–0.040	0.966
*Growth*	U	2.618	3.011	–6.0	81.30	–1.090	0.275
	M	2.663	2.589	1.1		0.220	0.829
*Inv*	U	0.477	0.531	–24.1	94.90	–4.380	0.000
	M	0.486	0.483	1.2		0.220	0.827
*Loss*	U	0.075	0.058	6.7	–34.50	1.220	0.223
	M	0.074	0.097	–9.0		–1.440	0.149

Notes: U= Unmatched, M= Matched. %bias represents the standardized difference. %reduct ∣ bias ∣ shows the postmatching reduction in the absolute standardized mean difference. The t-test evaluates the statistical significance of the %bias.

**Table 6:** Endogeneity tests II.

Variables	PSM	Lagged Independent Variable
	(1)	(2)
	*Risk*	*Risk*
*LEVM*	0.169^**^	
	(2.368)	
LEVM_lag		0.140^***^
		(3.203)
*Size*	-0.505^***^	-0.311^*^
	(-3.117)	(-2.496)
*Liquid*	-0.445^***^	-0.432^***^
	(-6.397)	(-4.917)
*Msl*	6.239^***^	4.608^*^
	(2.634)	(2.319)
*Indep*	0.008	0.001
	(0.390)	(0.071)
*TobinQ*	-0.422^**^	-0.126
	(-2.343)	(-0.979)
*Roa*	-21.171^***^	-22.902^***^
	(-15.945)	(-12.007)
*Soe*	-0.410^**^	-0.263^*^
	(-2.139)	(-1.779)
*Board*	-0.619	-0.099
	(-0.929)	(-0.239)
*Dual*	0.086	0.051
	(0.865)	(0.474)
*Growth*	-0.003	0.003
	(-0.370)	(0.537)
*Inv*	0.680^*^	0.896^***^
	(1.941)	(3.048)
*Loss*	-0.564^***^	-0.568^***^
	(-4.241)	(-5.387)
*Constant*	7.160^*^	1.203
	(1.747)	(0.443)
Year FE	Yes	Yes
Firm FE	Yes	Yes
Observations	994	1210
Adj. R^2^	0.570	0.586

#### 5.3.4 Lagging the independent variables by one period.

To mitigate reverse causality concerns and account for the extended project cycles inherent to the real estate sector, where the financial risk implications of leverage manipulation may exhibit temporal delays, we apply a one-period lag to the core explanatory variable (*LEVM*), denoted as LEVM_lag. Column (2) in Table 6 reports the regression results using this lagged specification, showing a significantly positive coefficient of 0.140 for LEVM_lag at the 1% level. This finding confirms that the positive relationship between leverage manipulation and financial risk persists even after addressing endogeneity from bidirectional causality, thereby robustly supporting Hypothesis H1.

### 5.4 Robustness test

#### 5.4.1 Replacing the measurement of the dependent variable.

To further validate the findings, this study incorporates the O-Score model risk coefficient (Risk_C), constructed following Ohlson’s (1980) [73] methodology, as an alternative measure of financial risk.

$$Risk\_C=\frac{e^{O-Score}}{1+e^{O-Score}}$$ (6)

As reported in column (1) of Table 7, the coefficient for leverage manipulation remains statistically significant at the 1% level with a positive sign, corroborating the baseline conclusion that leverage manipulation exacerbates financial risk. This consistency across alternative risk specifications underscores the robustness of the primary findings.

**Table 7:** Robustness tests I.

Variables	(1)	(2)	(3)	(4)
	*Risk_C_*	*Risk*	*Risk*	*Risk*
*LEVM*	0.000^***^			0.269^***^
	(3.265)			(5.117)
*ExpLEVM*		0.203^***^		
		(2.958)		
*ExpLEVMI*			0.199^***^	
			(3.743)	
*Size*	-0.000^*^	-0.473^***^	-0.464^***^	-0.488^***^
	(-1.884)	(-3.402)	(-3.312)	(-2.816)
*Liquid*	-0.000^***^	-0.420^***^	-0.428^***^	-0.410^***^
	(-3.978)	(-4.909)	(-4.977)	(-3.591)
*Msl*	0.000	7.213^***^	7.048^***^	5.350^**^
	(0.224)	(2.992)	(2.877)	(2.200)
*Indep*	0.000	0.009	0.009	0.020
	(0.272)	(0.689)	(0.676)	(1.582)
*TobinQ*	0.000	-0.367^**^	-0.364^**^	-0.351^*^
	(0.721)	(-2.341)	(-2.307)	(-1.957)
*Roa*	-0.025^***^	-23.224^***^	-23.287^***^	-24.215^***^
	(-6.852)	(-13.047)	(-13.253)	(-10.290)
*Soe*	-0.001^**^	-0.365^**^	-0.354^**^	-0.333
	(-2.314)	(-2.310)	(-2.270)	(-1.617)
*Board*	0.000	-0.349	-0.346	-0.392
	(0.420)	(-0.724)	(-0.718)	(-0.599)
*Dual*	-0.000	0.006	0.001	-0.079
	(-0.565)	(0.059)	(0.011)	(-0.580)
*Growth*	-0.000	-0.000	-0.000	0.001
	(-1.552)	(-0.019)	(-0.043)	(0.089)
*Inv*	-0.000	0.865^***^	0.851^***^	0.885^**^
	(-0.460)	(2.663)	(2.640)	(2.235)
*Loss*	-0.000	-0.589^***^	-0.583^***^	-0.800^***^
	(-0.611)	(-4.978)	(-4.978)	(-4.811)
*Constant*	0.010^***^	5.626	5.425	5.682
	(2.814)	(1.629)	(1.563)	(1.242)
Year FE	Yes	Yes	Yes	Yes
Firm FE	Yes	Yes	Yes	Yes
Observations	1316	1316	1316	942
Adj. R^2^	0.575	0.544	0.549	0.576

#### 5.4.2 Replacing the measurement of the independent variable.

To verify the robustness of our findings, we recalculate the leverage manipulation levels of real estate firms using the expanded XLT-LEVM method under both direct and indirect approaches, substituting the core explanatory variable. As shown in Table 7, columns (2) and (3) present results based on the two variants. Column (2) reports a significantly positive coefficient of 0.203 for *ExpLEVM* at the 1% level, while column (3) shows a coefficient of 0.199 for *ExpLEVMI*, also significant at the 1% level. These results confirm the persistent risk-amplifying effect of leverage manipulation.

#### 5.4.3 Excluding COVID-19’s exogenous shock.

Wind data reveals that Chinese real estate bond defaults surged by 533% year-on-year to 28.17 billion yuan in 2020, primarily among mid-sized developers. To isolate the exogenous impact of COVID-19, we exclude observations from 2020 and re-estimate the model using the 2009–2019 sample. Column (4) of Table 7 indicates that the coefficient for *LEVM* remains statistically significant at the 1% level with a positive sign, reinforcing the baseline conclusion.

#### 5.4.4 Adding macro-level control variables.

Given real estate’s heightened policy sensitivity and regional heterogeneity, we augment Eq (5) with city-level controls: logged GDP per capita (*Lrgdp*), financial development (*Gloan*), and population density (*Lpopi*). Column (1) in Table 8 reports a significantly positive coefficient for *LEVM* at the 1% level, indicating robustness to regional economic factors.

**Table 8:** Robustness tests II.

Variables	(1)	(2)	(3)
	*Risk*	*Risk*	*Risk*
*LEVM*	0.204^***^	0.219^***^	0.204^***^
	(2.909)	(2.984)	(2.820)
*Size*	-0.477^***^	-0.512^***^	-0.474^***^
	(-3.410)	(-3.179)	(-3.989)
*Liquid*	-0.420^***^	-0.406^***^	-0.420^***^
	(-4.880)	(-3.710)	(-6.126)
*Msl*	7.230^***^	4.414	7.218^***^
	(2.997)	(1.483)	(3.559)
*Indep*	0.009	0.022	0.009
	(0.693)	(1.129)	(0.680)
*TobinQ*	-0.368^**^	-0.207	-0.368^***^
	(-2.303)	(-1.147)	(-2.818)
*Roa*	-23.256^***^	-24.164^***^	-23.225^***^
	(-13.034)	(-10.233)	(-15.388)
*Soe*	-0.363^**^	-0.505^**^	-0.365^*^
	(-2.330)	(-2.271)	(-2.119)
*Board*	-0.338	-0.038	-0.349
	(-0.713)	(-0.070)	(-0.702)
*Dual*	0.003	0.043	0.006
	(0.029)	(0.383)	(0.073)
*Growth*	-0.000	0.002	-0.000
	(-0.045)	(0.308)	(-0.017)
*Inv*	0.876^***^	1.291^***^	0.864^**^
	(2.684)	(3.619)	(2.619)
*Loss*	-0.590^***^	-0.672^***^	-0.589^***^
	(-4.928)	(-4.231)	(-4.191)
*Lrgdp*	-0.104		
	(-0.286)		
*Gloan*	-0.041		
	(-0.246)		
*Lpopi*	0.000		
	(0.083)		
*Constant*	6.925	5.160	5.630
	(1.137)	(1.534)	(1.555)
Year FE	Yes	Yes	Yes
Firm FE	Yes	Yes	Yes
City-Year FE	No	No	Yes
Observations	1296	1101	1316
Adj. R^2^	0.542	0.560	0.544

*Note:* Due to missing observations of macroeconomic indicators for some prefecture-level cities in 2023, column (1) has fewer observations than the full sample. Additionally, in column (2), the t-values in parentheses indicate cluster-robust standard errors at the city level.

#### 5.4.5 City-level cluster analysis.

Given that real estate firms’ operational decisions are constrained by factors such as purchase restrictions, land supply cycles, and credit availability, unobserved intra-city correlations may exist among developers within the same municipality. To address potential heteroskedasticity and serial correlation, this study adjusts standard errors from firm-level clustering to city-level clustering. As shown in column (2) of Table 8, the coefficient for leverage manipulation (*LEVM*) remains statistically significant and positive at the 1% level, with no material changes in significance or directionality.

#### 5.4.6 Adding city-year interaction terms.

To more accurately control for potential disturbances, we added city-year interaction terms to the model. Column (3) of Table 8 shows the results after including these terms. The *LEVM* coefficient remains significantly positive at the 1% level, indicating that the original conclusion is robust.

## 6 Further analysis

The preceding empirical results preliminarily validate the study’s hypotheses, demonstrating that leverage manipulation exacerbates financial risks in real estate firms. Building on the interest conflict perspective, this section delves into the mechanisms through which stakeholders influence the relationship between leverage manipulation and financial risk, focusing on two dimensions: (1) the moderating roles of major shareholders and external auditing and (2) heterogeneity analyses based on ownership type, institutional investor heterogeneity, and regional land-finance dependency.

### 6.1 Mechanism analysis

#### 6.1.1 Major shareholders.

To further examine how controlling shareholders’ conflicts of interest influence the extent of corporate leverage manipulation, this study uses the ownership share of the largest shareholder (*Top*1) to measure ownership concentration. Specifically, based on the Eq (5), an interaction term between leverage manipulation and ownership concentration (LEVM×Top1) is added to construct Eq (7), which is used to test hypotheses H2a and H2b. Suppose the coefficient of *LEVM* is significantly positive, and the interaction term LEVM×Top1 also shows a significantly positive effect. In that case, it suggests the presence of a collusion effect between controlling shareholders and management, which amplifies the impact of leverage manipulation on financial risk. Conversely, if the coefficient of LEVM×Top1 is significantly negative, it implies that controlling shareholders play a monitoring role and effectively mitigate the positive relationship between leverage manipulation and financial risk.

To ensure the robustness of the analysis, we further re-estimate the degree of collusion using an alternative interaction term between leverage manipulation and the combined shareholding of the top three shareholders (LEVM×Top3). In addition, to address potential multicollinearity between the interaction terms and their respective main effects, all continuous moderating and independent variables are mean-centered prior to estimation.

RISKi,t=β0+β1LEVMi,t+β2LEVM×Top1(Top3)i,t+β3Top1(Top3)i,t=+β4Controlsi,t+μi,t+ηi,t+εi,t (7)

As shown in Table 9, after introducing the interaction term between leverage manipulation and the controlling shareholder, the coefficient of the core explanatory variable (*LEVM*) decreases from 0.204 to 0.145, indicating that controlling shareholders partially absorb the impact of leverage manipulation on financial risk. Meanwhile, the coefficient of the interaction term LEVM×Top1 is 1.082 and is significantly positive at the 10% level. In the robustness test, the coefficient of LEVM×Top3 is significantly positive at the 1% level. These findings indicate a significant collusive reinforcement mechanism between controlling shareholders and leverage manipulation, providing empirical support for Hypothesis H2b.

**Table 9:** Moderating effect test I.

Variables	(1)	(2)	(3)
	*Risk*	*Risk*	*Risk*
*LEVM*	0.204^***^	0.145^***^	0.094^***^
	(2.942)	(2.636)	(3.666)
LEVM×Top1		1.082^*^	
		(1.981)	
*Top*1		1.334^***^	
		(3.057)	
LEVM×Top3			0.016^***^
			(5.593)
*Top*3			0.009
			(1.510)
*Size*	-0.474^***^	-0.490^***^	-0.457^***^
	(-3.403)	(-3.586)	(-3.225)
*Liquid*	-0.420^***^	-0.433^***^	-0.463^***^
	(-4.908)	(-5.121)	(-5.390)
*Msl*	7.218^***^	7.089^***^	5.917^**^
	(2.996)	(2.944)	(2.315)
*Indep*	0.009	0.007	0.003
	(0.690)	(0.582)	(0.248)
*TobinQ*	-0.368^**^	-0.315^**^	-0.308^*^
	(-2.342)	(-2.022)	(-1.974)
*Roa*	-23.225^***^	-23.517^***^	-23.374^***^
	(-13.045)	(-13.218)	(-12.769)
*Soe*	-0.365^**^	-0.240	-0.294^**^
	(-2.311)	(-1.615)	(-2.024)
*Board*	-0.349	-0.364	-0.385
	(-0.723)	(-0.788)	(-0.821)
*Dual*	0.006	-0.005	0.014
	(0.058)	(-0.048)	(0.139)
*Growth*	-0.000	-0.001	-0.001
	(-0.019)	(-0.230)	(-0.233)
*Inv*	0.864^***^	0.826^**^	0.838^***^
	(2.660)	(2.585)	(2.683)
*Loss*	-0.589^***^	-0.577^***^	-0.578^***^
	(-4.976)	(-4.848)	(-4.911)
*Constant*	5.630	6.063^*^	5.562
	(1.630)	(1.745)	(1.539)
Year FE	Yes	Yes	Yes
Firm FE	Yes	Yes	Yes
Observations	1316	1316	1316
Adj. R^2^	0.544	0.553	0.570

#### 6.1.2 External auditing.

This study employs positive abnormal audit fees (*HIABFEE*) as a proxy for the external audit’s dual signaling role, aligning with the hypothesis of stakeholder interest conflicts. Positive abnormal fees encapsulate two conflicting signals: a “monitoring premium” reflecting heightened audit scrutiny and a “collusion consideration” indicating auditor-client collusion [81]. On the one hand, audit firms may allocate experienced teams and exert additional effort to mitigate litigation risks and reputational damage, justifying higher fees through rigorous risk assessment and response protocols. On the other hand, excessive fees may signal compromised independence, where auditors tolerate leverage manipulation to retain lucrative clients [82].

Following Blankley and Hurtt. (2012) and Doogar et al. (2015) [83,84], we construct an audit pricing Eq (8) to estimate abnormal audit fees (*ABFEE*). To isolate the impact of excessive audit fees, we create a binary variable (*HIABFEE*) that equals 1 if the residual from the audit fee model (*ABFEE*) is strictly positive, indicating fees higher than expected based on firm characteristics; otherwise, it equals 0. The audit fee model includes the following control variables: accounts receivable and inventory ratios (*ARInv*), leverage ratio (*Lev*), square root of employees (*Employ*), audit lag (*Delay*), auditor size (*Officesize*), Big Four affiliation (*BIG*4), domestic Top 8 affiliation (*Tier*2), auditor changes (*Change*), year fixed effects (*η*), and industry fixed effects (*θ*).

$${LNFEE}_{i,t}=\lambda_{0}+\lambda_{1}{Size}_{i,t}+\lambda_{2}{ARInv}_{i,t}+\lambda_{3}{CATA}_{i,t}+\lambda_{4}{Liquid}_{i,t}+\lambda_{5}{Roa}_{i,t}\;\;+\lambda_{6}{Lev}_{i,t}+\lambda_{7}{Loss}_{i,t}+\lambda_{8}{Employ}_{i,t}+\lambda_{9}{BIG4}_{i,t}+\lambda_{10}{Tier2}_{i,t}\;\;+\lambda_{11}{Officesize}_{i,t}+\lambda_{12}{Change}_{i,t}+\lambda_{13}{Delay}_{i,t}+\eta_{i,t}+\theta_{i,t}+\varepsilon_{i,t}$$ (8)

To test these competing hypotheses, Eq (9) extends Eq (5) by incorporating interaction terms between leverage manipulation (*LEVM*) and audit characteristics. Following Gul et al. (2013) [85], robustness is assessed using audit quality (*ARAgg*), calculated as *ARAgg* = *Opinion* - *MAOs*, where *Opinion* equals 1 for modified audit opinions and 0 otherwise, and *MAOs* represent the predicted probability of modified opinions. Higher *ARAgg* values indicate greater audit quality, reflecting auditors’ adherence to standards.

$${RISK}_{i,t}=\beta_{0}+\beta_{1}{LEVM}_{i,t}+\beta_{2}{LEVM\;\times \;ABFEE(ARAgg)}_{i,t}\;\;+\gamma_{3}{ABFEE(ARAgg)}_{i,t}+\gamma_{4}{Controls}_{i,t}+\mu_{i,t}+\eta_{i,t}+\varepsilon_{i,t}$$ (9)

As presented in Table 10, the coefficient for leverage manipulation (*LEVM*) increases from 0.204 to 0.210 (significant at the 1% level) after incorporating the interaction term between external audit and leverage manipulation (LEVM×HIABFEE). In contrast, the interaction term itself shows a significantly positive coefficient of 1.306 at the 1% level. Column (2) further reports robustness checks, where both *LEVM* and the alternative interaction term, LEVM×ARAgg, remain significantly positive, corroborating the collusion hypothesis in H3b.

**Table 10:** Moderating effect test II.

Variables	(1)	(2)
	*Risk*	*Risk*
*LEVM*	0.210^***^	0.160^***^
	(3.740)	(4.301)
LEVM×HIABFEE	1.306^***^	
	(4.842)	
*HIABFEE*	0.050	
	(0.132)	
LEVM×ARAgg		2.182^***^
		(3.967)
*ARAgg*		-0.669^*^
		(-1.793)
*Size*	-0.459^***^	-0.442^***^
	(-3.505)	(-3.218)
*Liquid*	-0.484^***^	-0.490^***^
	(-6.987)	(-7.340)
*Msl*	7.307^***^	7.096^***^
	(2.944)	(2.838)
*Indep*	0.008	0.010
	(0.692)	(0.759)
*TobinQ*	-0.344^**^	-0.431^***^
	(-2.263)	(-2.978)
*Roa*	-21.972^***^	-20.870^***^
	(-14.636)	(-16.899)
*Soe*	-0.326^**^	-0.281^*^
	(-2.031)	(-1.865)
*Board*	-0.161	-0.376
	(-0.350)	(-0.820)
*Dual*	0.023	0.024
	(0.218)	(0.230)
*Growth*	-0.002	-0.001
	(-0.323)	(-0.201)
*Inv*	0.860^***^	0.935^***^
	(2.795)	(2.862)
*Loss*	-0.588^***^	-0.607^***^
	(-4.765)	(-5.008)
*Constant*	4.994	5.029
	(1.461)	(1.473)
Year FE	Yes	Yes
Firm FE	Yes	Yes
Observations	1275	1302
Adj. R^2^	0.585	0.560

### 6.2 Heterogeneity analysis

#### 6.2.1 Property rights nature.

The corporate ownership structure has a significant influence on a firm’s financial risk exposure. Theoretically, firms facing tighter financing constraints exhibit more substantial incentives for leverage manipulation. Compared to non-state-owned real estate enterprises (Non-SOEs), state-owned counterparts (SOEs) demonstrate pronounced advantages in economies of scale, typically operating at larger organizational sizes. Additionally, SOEs benefit from lower credit barriers due to implicit government guarantees [86], resulting in weaker financing constraints and reduced motives for leverage manipulation. Non-SOEs, which are constrained by limited policy support and financing channels, prioritize reducing their debt-to-asset ratio to signal financial stability and attract long-term strategic investments [87]. We thus posit that leverage manipulation exerts more substantial risk-amplifying effects among Non-SOEs relative to SOEs.

Table 11 reports the heterogeneity analysis by enterprise ownership. Grouped regressions in Columns (1) and (2) show that the leverage manipulation (*LEVM*) coefficient for non-state-owned real estate firms is 0.287, significantly positive at the 1% level. In contrast, the coefficient for state-owned enterprises (0.085) does not reach statistical significance. The notable magnitude difference (0.287 vs. 0.085) highlights a more pronounced risk-amplifying effect of leverage manipulation in non-state-owned firms.

**Table 11:** Heterogeneity test I.

Variables	SOEs	Non-SOEs
	(1)	(2)
	*Risk*	*Risk*
*LEVM*	0.085	0.287^***^
	(1.541)	(7.658)
*Size*	-0.393^**^	-0.499^*^
	(-2.026)	(-2.011)
*Liquid*	-0.441^***^	-0.454^***^
	(-5.117)	(-3.284)
*Msl*	4.623^***^	5.905^*^
	(4.390)	(1.815)
*Indep*	0.018	-0.006
	(0.929)	(-0.220)
*TobinQ*	-0.138	-0.350
	(-0.653)	(-1.364)
*Roa*	-22.640^***^	-24.339^***^
	(-12.306)	(-8.085)
*Board*	0.439	-1.384^*^
	(0.771)	(-1.695)
*Dual*	0.095	0.019
	(0.789)	(0.113)
*Growth*	0.003	-0.006
	(0.910)	(-0.338)
*Inv*	0.632^*^	0.961
	(1.738)	(1.601)
*Loss*	-0.469^***^	-0.663^***^
	(-3.677)	(-2.948)
*Constant*	1.487	8.614
	(0.410)	(1.181)
Year FE	Yes	Yes
Firm FE	Yes	Yes
Observations	795	501
Adj. R^2^	0.597	0.532

The absence of significance in state-owned enterprises can be attributed to institutional traits that mitigate the direct propagation of risk. Their ability to obtain government-endorsed implicit guarantees provides stable financing avenues, thereby lessening the need for leverage manipulation to address liquidity needs. Simultaneously, the administrative governance mechanisms within state-owned systems partially detach managerial risk-taking from immediate corporate insolvency costs, allowing off-balance-sheet liabilities to be reallocated through intra-group transactions rather than emerging as explicit risks in individual financial reports. It is crucial to emphasize that this short-term lack of statistical significance does not imply the absence of risk; instead, systemic risks may gradually accumulate through cross-guarantee networks or inter-enterprise financial linkages within the state-owned sector, potentially resulting in delayed yet more concentrated risk manifestations that go beyond the scope of single-firm financial disclosures.

#### 6.2.2 Heterogeneous institutional investors.

Institutional investors exert dual influences in capital markets, with their inherent conflicts of interest profoundly shaping corporate financial decisions and risk transmission. While institutions possess informational and analytical advantages over retail investors [88], their governance impact is not unidirectional but contingent on profit-seeking motives. From a long-term alignment perspective, value-oriented institutions translate expertise into active oversight—intervening in governance, vetoing high-risk financing schemes, and identifying leverage manipulation tactics such as off-balance-sheet liabilities and non-share-real-debts. However, principal-agent conflicts inherent in modern corporations may distort institutional roles [89]. When short-term arbitrage dominates, institutions may collude with management or major shareholders to engage in earnings management and financial window dressing, creating an illusion of performance while obscuring accurate risk exposures. This strategic collusion fosters a hidden accumulation of leverage-driven financial risks. Consequently, we posit that transient institutional investors exacerbate the risk-amplifying effects of leverage manipulation compared to stable, long-term-oriented institutions.

Following An and Zhang. (2013) and Wang et al. (2020) [90,91] classify institutions into stable and transactional types. As shown in Table 12, column (1) reveals that the coefficient for leverage manipulation (*LEVM*) in the stable institutional investor group is -0.060, significant at the 5% level. In contrast, Column (2) shows a positive coefficient of 0.047, which is also significant at the 5% significance level for the transactional group, aligning with theoretical expectations. The negative coefficient for stable institutions reflects their active governance mechanisms, such as blocking high-risk off-balance-sheet financing proposals and mandating third-party assessments of implicit liabilities, which identify and rectify leverage-induced distortions, thereby weakening the transmission of risk. These interventions partially offset the risk-amplifying effects of leverage manipulation in firms dominated by stable institutions.

**Table 12:** Heterogeneity test II.

Variables	Stable-Inst	Transient-Inst
	(1)	(2)
	*Risk*	*Risk*
*LEVM*	-0.060^**^	0.047^**^
	(-2.425)	(2.286)
*Size*	-0.229^*^	-0.153
	(-1.819)	(-1.085)
*Liquid*	-0.546^***^	-0.333^**^
	(-7.712)	(-2.453)
*Msl*	1.871^*^	2.209
	(1.988)	(1.320)
*Indep*	0.004	0.017
	(0.551)	(1.050)
*TobinQ*	0.101	-0.069
	(0.713)	(-0.428)
*Roa*	-22.728^***^	-22.195^***^
	(-15.387)	(-7.272)
*Soe*	-0.023	-0.347
	(-0.169)	(-1.245)
*Board*	0.291	-0.138
	(0.804)	(-0.251)
*Dual*	-0.115	-0.027
	(-1.190)	(-0.152)
*Growth*	0.007^*^	0.005
	(1.978)	(0.606)
*Inv*	1.041^***^	1.265^***^
	(4.155)	(3.168)
*Loss*	-0.598^***^	-0.420^***^
	(-5.296)	(-2.803)
*Constant*	-1.920	-3.484
	(-0.703)	(-1.123)
Year FE	Yes	Yes
Firm FE	Yes	Yes
Observations	660	609
Adj. R^2^	0.816	0.671

#### 6.2.3 Land financial dependence.

The core conflict of interest for local governments lies in their dual roles as both promoters of growth and enforcers of deleveraging. On the one hand, the entrenched reliance on land finance has led to widespread regulatory leniency toward real estate firms, which aim to boost local GDP. On the other hand, China’s mandatory deleveraging campaign, initiated in late 2015, prioritizes corporate debt reduction, requiring state-owned enterprises to reduce their leverage ratios by 2% by 2020. Under the current fiscal system, land sales revenue serves as a crucial source of funding for addressing local deficits and investing in infrastructure. This dependency, exacerbated by officials’ tenure-driven incentives, fosters a short-term “land-for-growth” logic: local governments manipulate land supply to maximize revenue, driving up land prices that inflate housing costs, which in turn incentivize speculative land acquisitions by developers [92].

While this model temporarily accelerates GDP and fiscal revenue growth [93], it amplifies dual risks in local government and corporate debt. Local implicit guarantees blur market-government risk boundaries, encouraging financial institutions to relax lending standards under “rigid repayment” expectations. Conversely, central deleveraging mandates force banks to scrutinize developers’ reported liabilities, creating a regulatory paradox. Developers thus engage in leverage manipulation to meet local land payment demands while evading central oversight, neutralizing both short-term local growth objectives and long-term systemic risk prevention. Consequently, this study posits that firms in low land finance dependency regions face higher financing costs, thereby amplifying the risk-enhancing effects of leverage manipulation compared to those in high land finance dependency regions.

Following Tian and Ma (2009) and Ye and Wu (2014) [94,95], land finance dependency (*LDF*) is proxied by the ratio of municipal land sales revenue to general fiscal revenue. Samples are stratified into low-LDF, medium-LDF, and high-LDF groups based on annual terciles.

As presented in Table 13, Column (1) reports the regression results for the low land fiscal dependence group, where the coefficient of leverage manipulation is 0.297, statistically significant at the 1% level. In contrast, the coefficient for the high land fiscal dependence group is 0.11 and insignificant. Notably, despite the insignificant result in the high-dependence subgroup, the coefficient in the low-dependence group is nearly 2.7 times larger than that in the high-dependence group (0.297 vs. 0.11). This magnitude difference suggests that when local governments rely less on land revenue, corporate leverage manipulation exerts a more pronounced impact on financial risk, which aligns with our prior hypothesis.

**Table 13:** Heterogeneity test III.

Variables	Low-LFD	High-LFD
	(1)	(2)
	*Risk*	*Risk*
*LEVM*	0.296^***^	0.111
	(4.037)	(1.251)
*Size*	-0.387	-0.655^**^
	(-1.600)	(-2.334)
*Liquid*	-0.397^**^	-0.524^***^
	(-2.353)	(-3.646)
*Msl*	4.846	7.014^**^
	(0.855)	(2.173)
*Indep*	0.054^**^	-0.033
	(2.126)	(-1.384)
*TobinQ*	-0.102	-0.583
	(-0.389)	(-1.497)
*Roa*	-25.934^***^	-22.214^***^
	(-7.193)	(-7.672)
*Soe*	-0.759^***^	-0.266
	(-2.996)	(-1.013)
*Board*	0.357	-2.154^***^
	(0.555)	(-2.773)
*Dual*	-0.140	0.002
	(-0.790)	(0.013)
*Growth*	0.009	-0.002
	(1.219)	(-0.222)
*Inv*	0.479	1.417^**^
	(0.994)	(2.260)
*Loss*	-1.086^***^	-0.406^**^
	(-3.930)	(-2.103)
*Constant*	0.567	15.450^*^
	(0.117)	(1.810)
Year FE	Yes	Yes
Firm FE	Yes	Yes
Observations	435	429
Adj. R^2^	0.537	0.429

A plausible explanation for the insignificant high-dependence coefficient may relate to institutional buffers in regions with strong land fiscal ties: governments in these areas often provide implicit guarantees or preferential financing to real estate firms, potentially mitigating the direct risk transmission from leverage manipulation. By contrast, in low-dependence regions, firms face fewer policy subsidies and tighter market discipline, making their financial fragility more sensitive to leverage distortions. While statistical significance is absent in the high-dependence group, the clear directional difference in coefficients supports the theoretical prediction that land fiscal reliance weakens the risk-amplifying effect of leverage manipulation.

## 7 Conclusion and limitations

### 7.1 Conclusion

This study systematically examines the mechanisms and constraints of leverage manipulation on financial risk among Chinese A-share-listed real estate firms from 2009 to 2023, grounded in an interest conflict perspective. The findings reveal that leverage manipulation significantly amplifies corporate financial risks. Further analysis demonstrates pervasive conflicts of interest among stakeholders. Specifically, mechanism tests indicate collusion between major shareholders and management, as well as between external auditors and management, which exacerbates the risk-enhancing effects of leverage manipulation. Heterogeneity analyses show that the adverse impacts of leverage manipulation are more pronounced in private developers and firms operating in regions with lower land finance dependency. In contrast, stable institutional investors, compared to transactional counterparts, mitigate risk transmission by actively engaging in governance to identify and correct financial distortions.

### 7.2 Limitation and future research

This study acknowledges several limitations. First, although the research provides insights into leverage manipulation in China’s real estate sector, its generalizability to other high-leverage industries, which include infrastructure and energy, is constrained by significant disparities in institutional frameworks and risk propagation mechanisms. Second, the analysis overlooks the influence of key stakeholders, such as suppliers and homebuyers, whose contractual and financing practices can indirectly modulate leverage dynamics. For example, while extended supplier credit terms may obscure short-term liquidity risks, presale deposits from homebuyers create contingent liabilities that interact with off-balance-sheet financing, thereby amplifying systemic vulnerabilities. Future research could address these gaps through cross-sectoral comparative analyses and the integration of multi-stakeholder interaction models.

## Supporting information

S1 Dofile The analysis code file includes structured commands for summary statistics, fixed-effects regressions, robustness tests, heterogeneity analysis, and figure generation. It enables full replication of the empirical procedures and results reported in the manuscript.
(TXT)

S1 Dataset Firm-level panel data used in baseline regression, mechanism tests, and heterogeneity analysis. Financial data are sourced from the CSMAR Database (https://data.csmar.com/) under institutional license. Prefectural-level macroeconomic data are accessible via the National Bureau of Statistics of China (https://www.stats.gov.cn/) under the “Regional Data” section. No additional ethical approval was required, as all data are anonymized and publicly available.
(XLSX)

S2 Dataset Annual averages of housing prices and leverage ratios in China’s real estate sector from 2009 to 2023. This dataset supports the time-series visualization presented in Fig 1 and is formatted to facilitate replication and inspection.
(XLSX)
